# Influence of surgical and N95 face masks on speech perception and listening effort in noise

**DOI:** 10.1371/journal.pone.0253874

**Published:** 2021-07-01

**Authors:** Torsten Rahne, Laura Fröhlich, Stefan Plontke, Luise Wagner

**Affiliations:** Department of Otorhinolaryngology, University Hospital Halle (Saale), Martin Luther University Halle-Wittenberg, Halle (Saale), Germany; VIT University, INDIA

## Abstract

Daily-life conversation relies on speech perception in quiet and noise. Because of the COVID-19 pandemic, face masks have become mandatory in many situations. Acoustic attenuation of sound pressure by the mask tissue reduces speech perception ability, especially in noisy situations. Masks also can impede the process of speech comprehension by concealing the movements of the mouth, interfering with lip reading. In this prospective observational, cross-sectional study including 17 participants with normal hearing, we measured the influence of acoustic attenuation caused by medical face masks (mouth and nose protection) according to EN 14683 and of N95 masks according to EN 1149 (EN 14683) on the speech recognition threshold and listening effort in various types of background noise. Averaged over all noise signals, a surgical mask significantly reduced the speech perception threshold in noise was by 1.6 dB (95% confidence interval [CI], 1.0, 2.1) and an N95 mask reduced it significantly by 2.7 dB (95% CI, 2.2, 3.2). Use of a surgical mask did not significantly increase the 50% listening effort signal-to-noise ratio (increase of 0.58 dB; 95% CI, 0.4, 1.5), but use of an N95 mask did so significantly, by 2.2 dB (95% CI, 1.2, 3.1). In acoustic measures, mask tissue reduced amplitudes by up to 8 dB at frequencies above 1 kHz, whereas no reduction was observed below 1 kHz. We conclude that face masks reduce speech perception and increase listening effort in different noise signals. Together with additional interference because of impeded lip reading, the compound effect of face masks could have a relevant impact on daily life communication even in those with normal hearing.

## Introduction

Coronavirus disease 2019 (COVID-19) is caused by infection with the novel SARS-CoV-2 coronavirus. The disease, which primarily affects the respiratory tract, was first described in Wuhan, China, at the end of 2019. It developed into an epidemic in that country in January 2020 and was declared a pandemic in March 2020. Evidence supports the potential for transmission in superspreading events [1, 2], and infection usually occurs through transmission of droplets. Aerosol transmission is possible, especially in closed, poorly ventilated rooms [3, 4].

In many countries, including Germany and the United States of America, regulatory bodies such as the Robert Koch Institute (RKI) and the U.S. Centers for Disease Control and Prevention (CDC) call for the use of mouth and nose protection or even a medical face mask [5] to reduce infection risk. For many social situations, local or regional rules have made this use mandatory.

Use of masks can raise obstacles to communication [6]. Masks cover the mouth, which impedes the gain of information from lip and facial movements to support aural comprehension. Lip reading is especially important for hearing in noisy situations because these movements provide temporal clues and increase awareness of the language elements. Particularly, information about spoken consonants is provided [7]. Access to lip reading cues has a positive effect on speech understanding in background noise, especially for people who have hearing impairment [8]. The speech reception threshold (SRT) in background noise can be improved by 3–5 dB if the face is visible [9, 10].

Masks also can affect the acoustic properties of the speech signal itself, with negative implications for speech perception. Simple medical masks, such as those used in operating rooms, reduce the spoken language level by 3–4 dB in the high-frequency range of 2000–7000 Hz, and N95 masks reduce the level by about 12 dB [11]. A decrease of 12 dB has also been measured for surgical masks [12]. Although speaking through face masks alters the speech signal, some specific features of voice quality features (e.g., harmonic-to-noise ratio, temporal pattern) remain largely unaffected [13]. The sound pressure level of spoken language is reduced mainly at frequencies ≥2000 Hz, so that the resulting signal would be similar to that for listeners having who have slight high-frequency hearing loss [14]. In a recent study of health workers, the speech perception threshold was increased by 12.4 dB if the speaker used an N95 mask and a face shield [15].

In listeners with normal hearing, face masks do not significantly affect speech intelligibility in noise [6, 16, 17]. However, studies making this assessment did not measure the speech perception threshold (SRT) in noise, i.e., the signal-to-noise ratio (SNR) that results in 50% speech recognition. SRT is widely used in the clinical routine as a sensitive diagnostic tool. In this study, we used the Oldenburg sentence test (OLSA), which presents lists of unpredictable five-word sentences (name—verb—numeral—adjective—object) to the listener in a background masker with different SNRs [18]. Depending on the number of words (score) correctly repeated by the listener, the SNR is increased or decreased for the subsequent sentence presentation. The adaptive procedure converges on the SNR that corresponds to a 50% correct score, defined as SRT_50_.

Even a little signal level attenuation, as expected with the use of face masks, would affect hearing in a noisy situation with SNRs close to the individual SRT. In hearing-impaired listeners, speech perception in noise would be more affected by face mask–related declines in sound pressure levels because of the reduced amplification by the outer hair cells, resulting in a smaller dynamic range [6, 8, 19, 20]. In hearing-impaired individuals, speech perception in noise also is reduced to an increased pure-tone threshold in quiet [21, 22], mainly attributable to a reduced cochlear amplifier function. Other than in conductive hearing loss, hearing aids can compensate only partially for impaired hair cell function. Therefore, reduced speech level in noise would particularly affect daily life communication in hearing-impaired listeners. For such “pseudo hearing impairment”, hearing amplifiers that compensate for these deficits are required, especially for vulnerable occupational groups and social groups [23]. Specific recommendations for educational settings also aim to compensate for the negative aspects of mouth and nose protection [24].

Active listening requires cognitive resources such as focus and attention. A decreased speech level in quiet or SNR in noisy situations increases stress on cognitive resources [25]. The use of mouth and nose protection also is expected to increase the listening effort, which in turn has consequences for many daily life situations with a high communication load, such as a classroom. With the Adaptive CAtegorical Listening Effort Scaling Test (ACALES), a clinical procedure has recently become available that enables measurement of the listening effort amid background noise [26]. Sentences are presented in a background masker with different SNRs. Subjective listening effort is quantified on a 14-point subjective categorical scale ranging from ‘no effort’ to ‘extreme effort’. SNRs of ACALES are adaptively adjusted for each presentation based on previous ratings, to individually cover the entire range of possible categories and to finally determine the absolute threshold (SNR_cut_) with a moderate listening effort [27, 28].

The aim of this research project was to measure the influence of acoustic attenuation caused by medical face masks (mouth and nose protection) according to EN 14683 and N95 masks according to EN 1149 (EN 14683) on the speech recognition threshold and the listening effort in various types of background noise in listeners with normal hearing.

## Materials and methods

This observational, cross-sectional study was conducted with adult volunteers with normal hearing. All participants were recruited by personnel contacts of the authors between November 2020 and January 2021 in Halle (Saale), Germany. Inclusion criteria were a minimum age of 18 years, normal hearing bilaterally, and fluency in German language (native speakers). Normal hearing was confirmed if the bilateral pure-tone thresholds for air conduction were ≤15 dB hearing level (HL) at both ears at the frequencies of 0.5, 1, 2, and 4 kHz. Audiological assessments were conducted using an AT900 audiometer (Auritec, Hamburg, Germany). Exclusion criteria were pregnancy and not meeting the inclusion criteria. This sample is considered representative of a larger population with normal hearing. Informed written consent was obtained from all participants for study inclusion. The study took place at the Audiology Lab of the University Hospital Halle (Saale), Germany, and was approved by the ethics committee of the Medical Faculty of the Martin Luther University Halle-Wittenberg (approval number 2020–160) and conducted in accordance with the Declaration of Helsinki.

**Fig 1:**
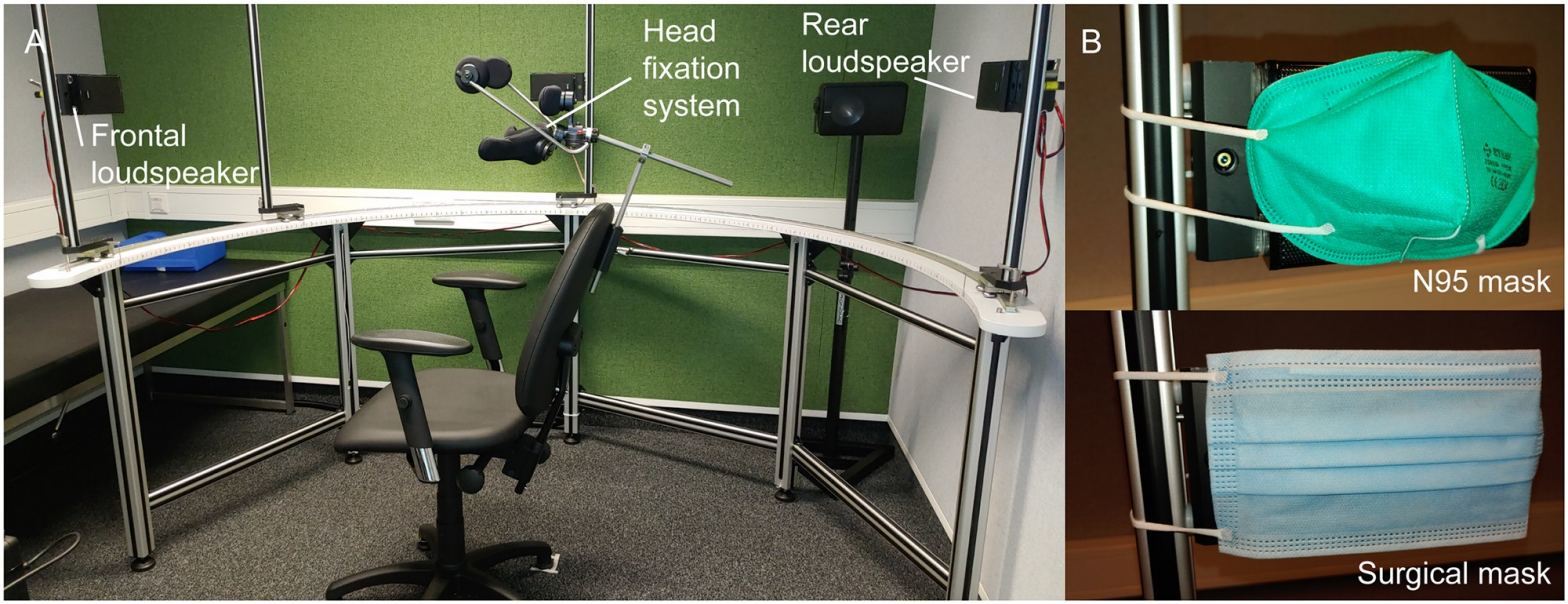
Experimental setup (A) and used face masks (B).

The influence of face masks on speech perception in noise was measured in a sound-attenuating booth using Foliodress LOOP TYPE IIR surgical face masks (CMC Medical Devices & Drugs, Malaga, Spain), according to European standard EN 14683 (‘surgical mask’), and RSN95B FFP2 NR particle-filtering half masks (Rysam Medical Equipment Manufacturing, Donguan City, China) (‘N95 mask’; Fig 1).

To measure the effects of masks on the acoustic features of the speech test signals, the masks were placed directly before the grid of a loudspeaker (CD 1020, Canton, Weilrod, Germany) which was positioned 1 m in front of a “dummy head” (KU 100, Neumann, Berlin, Germany; Fig 1). The olnoise speech simulation noise for a male speaker (‘Olnoise male’) and a female speaker (‘Olnoise female’) and the International Speech Test Signal (ISTS) [29] were presented for 30 s at a sound pressure level of 65 dB, using the Oldenburger Measurement Application 2.2 R&D software (Hörtech, Oldenburg, Germany), a Gigaport eX audio interface (ESI Audiotechnik, Leonberg, Germany), and a PLMRA400 amplifier (Pyle, Brooklyn, NY, USA). White noise was also presented for comparison. Sound signals were recorded by the microphones of the dummy head and amplified by a Fireface 400 audio interface (RME, Haimhausen, Germany). The root-mean-square (RMS) of the recording and the 1/3-octave amplitude spectrum were computed for every experimental condition by using Python scripts. The magnitude spectra of the two channels were averaged, and differences between recordings with and without masks were computed.

In listeners with normal hearing, speech recognition in noise was measured with the German Matrix Sentence Test OLSA (Hörtech, Oldenburg, Germany) using the same setup. Noise signals were continuously presented from behind at a sound pressure level of 60 dB. After two training runs, lists of 20 sentences were superposed and presented frontally (S_0_N_180_). The sound pressure level of every sentence was adjusted based on the participant’s response to the previous sentence to measure the open-set SRT for 50% correct recognition (SRT_50_) as the primary endpoint. ACALES v2.2 software (Hörtech, Oldenburg, Germany) was used to measure the listening effort. After two training runs, a series of two consecutive sentences with various SNRs from the OLSA in a 60-dB SPL background noise from a rear loudspeaker (S_0_N_180_) were frontally presented to the participants. Listening effort was measured in 14 effort categorical units (ecu). The speech level changed adaptively between −40 dB SNR and +20 dB SNR, based on the previous assessment of the subjectively perceived listening effort. Secondary endpoints were the SNR_cut_, i.e., the SNR at 7 ecu, and the slopes of the SNR-effort function for SNR with listening effort >7 ecu (m_low_) and <7 ecu (m_high_).

All participants were seated while the head was fixed using a Papillon head fixation system (Focal Meditech, Tilburg, The Netherlands; Fig 1). After two OLSA and two ACALES training runs, all participants completed the test runs while the noise signals (Olnoise female, Olnoise male, ISTS) and mask conditions (no mask, surgical mask, N95 mask) were applied in pseudorandomized order.

Primary and secondary endpoints were descriptively analyzed and tested for normality using the Shapiro–Wilk test. We then compared the distributions of SRT_50_ and SNR_cut_ with the results of an analysis of variance (ANOVA) for repeated measures using the within-subject factors of noise type (Olnoise female, Olnoise male, ISTS) and mask type (w/o mask, surgical mask, N95 mask). The assumption of sphericity was tested using Mauchly’s test, and we adjusted degrees of freedom with Bonferroni correction for all post-hoc comparisons. SPSS software version 25 (IBM, Ehningen, Germany) was used for statistical analyses.

## Results

Seventeen participants with normal hearing (14 female, 3 male) were included in the study. Their average age was 28 years (standard deviation [*SD*] = 5.4). The average pure-tone thresholds across participants and frequencies (4PTA_0.5-4kHz_) were 7.0 dB HL (*SD* = 2.8) for the left ear and 6.3 dB HL (*SD* = 3.2) for the right ear.

**Fig 2:**
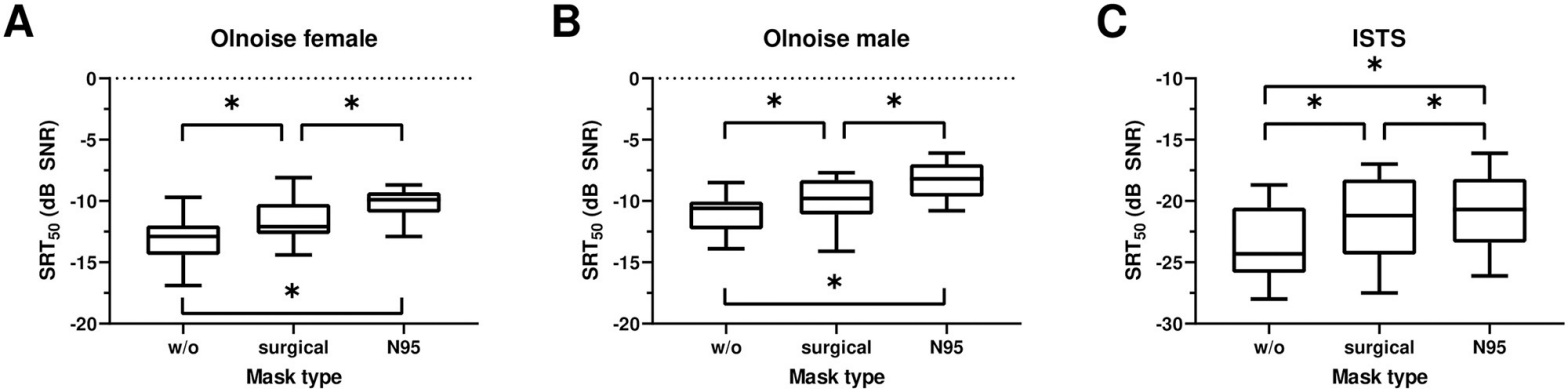
Distributions of speech perception in noise SRT_50_ for the Oldenburg Sentence Test (OLSA) presenting speech and noise signals from a female speaker (A) and a male speaker (B), or a female speaker in International Speech Test Signal (ISTS) (C). Putting a surgical face mask or an N95 mask between the speaker and the listener reduced the performance, as reflected by an increased SRT_50_. Boxes show the 25th, 50th and 75th percentiles, and whiskers indicate the 5th and 95th percentiles. Asterisks (*) indicate significant differences (*p* < 0.05).

Table 1 shows the descriptive data for the OLSA hearing-in-noise test and the ACALES listening effort test. Fig 2 shows the SRT_50_ distributions for all applied mask type conditions and noise signals. Mauchly’s test indicated that the assumption of sphericity was violated for the SRT_50_ (noise type: χ^2^(2) = 20.1, *p* < 0.001; mask type × noise type interaction: χ^2^(9) = 19.6, *p* < 0.05), so degrees of freedom were corrected using Greenhouse–Geisser estimates of sphericity. An ANOVA with noise type and mask type as within-subjects factors revealed main effects of mask type (*F*(1.9, 29.8) = 74.9, *p* < 0.001) and of noise type (*F*(1.2, 18.4) = 459.9, *p* < 0.001). We found no interaction between mask type and noise type (*F*(2.7, 42.5) = 1.2, *p* > 0.1). Post-hoc comparisons showed that a surgical mask increased the SRT_50_ significantly, by 1.6 dB (standard error [*SE*] = 0.21, confidence interval [*CI*] = 1.0, 2.1) on average. A further significant increase of 1.1 dB (*SE* = 0.25, *CI* = 0.5, 1.8) was detected with use of an N95 mask, resulting in a total increase of 2.7 dB (*SE* = 0.20, *CI* = 2.2, 3.2) across all noise signals. SRT_50_ was lowest for the ISTS (*M* = -21.8 dB, *SE* = 0.74), followed by the Olnoise female (*M* = -11.7 dB, *SE* = 0.35) and the Olnoise male (*M* = -9.7 dB, *SE* = 0.74) across all mask types.

**Table 1:** Speech perception in noise and listening effort results.

Mask type	Speech perception in noise			Listening effort					
	Olnoise male	Olnoise female	ISTS	Olnoise male			ISTS		
	Mean SRT_50_ (SD) [dB SNR]	Mean SRT_50_ (SD) [dB SNR]	Mean SRT_50_ (SD) [dB SNR]	Mean SNR_cut_ (SD) [escu]	Mean slope_low SNR_ (SD) [escu/dB]	Mean slope_high SNR_ (SD) [escu/dB]	Mean SNR_cut_ (SD) [escu]	Mean slope_low SNR_ (SD) [escu/dB]	Mean Slope_high SNR_ (SD) [escu/dB]
Without mask	-11.0 (1.6)	-13.2 (1.8)	-23.3 (2.9)	-6.3 (3.5)	-1.5 (1.0)	-1.1 (0.33)	-13.5 (6.0)	-0.83 (0.36)	-0.58 (0.16)
Surgical mask	-9.7 (1.6)	-11.6 (1.7)	-21.4 (3.4)	-5.4 (3.3)	-1.7 (1.2)	-1.1 (0.36)	-13.3 (5.2)	-0.91 (0.50)	-0.55 (0.12)
N95 mask	-8.3 (1.5)	-10.3 (1.2)	-20.7 (3.2)	-4.5 (3.5)	-1.7 (1.2)	-1.1 (0.27)	-11.1 (6.5)	-0.90 (0.78)	-0.68 (0.25)

ISTS: International Speech Test Signal; SD: standard deviation; SNR: signal-to-noise ratio; SRT_50_: 50% speech reception threshold.

**Fig 3:**
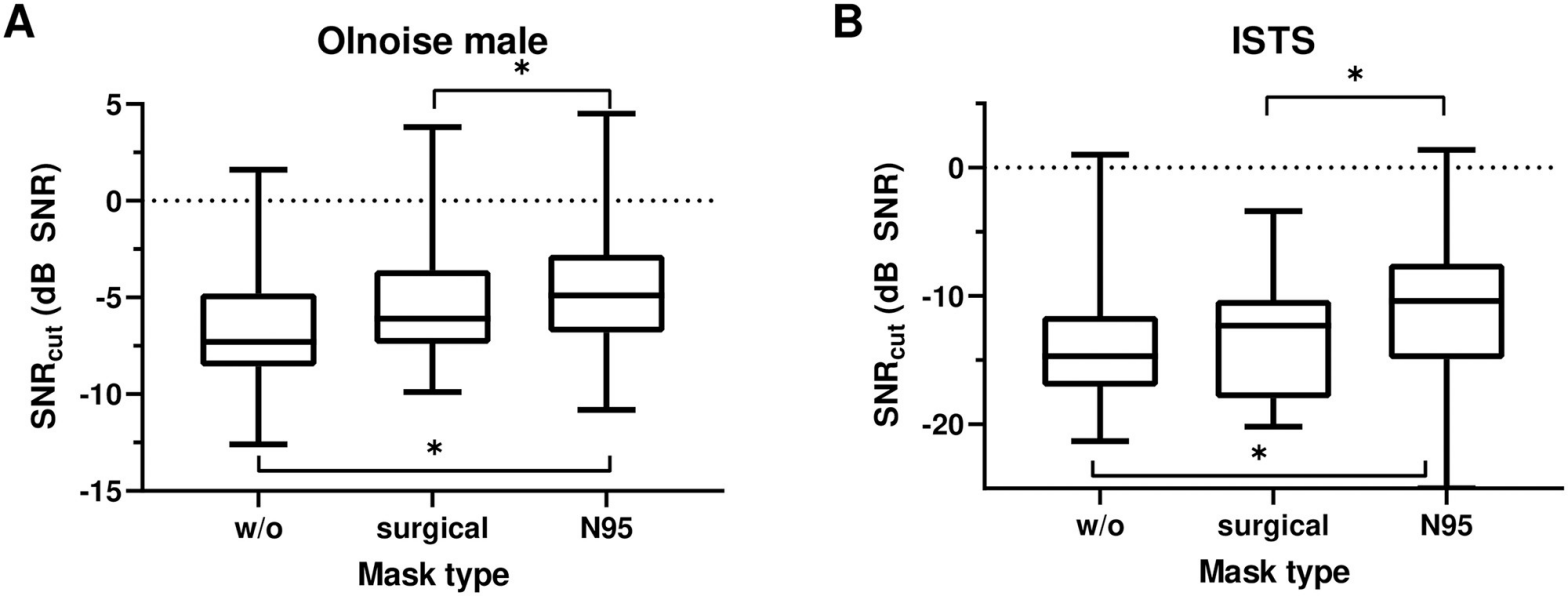
Distributions of listening effort in noise, SNR_cut_, for the ACALES presenting speech and noise signals from a male speaker (A) or a male speaker in International Speech Test Signal (ISTS) (B). Boxes show the 25th, 50th and 75th percentiles, and whiskers indicate the 5th and 95th percentiles. Asterisks (*) indicate significant differences (*p* < 0.05).

Fig 3 shows the SRT_cut_ distributions for all mask type conditions and noise signals. Mauchly’s test indicated that the assumption of sphericity had not been violated for the SNR_cut_ (*p* > 0.1). An ANOVA with noise type and mask type as within-subjects factors revealed main effects of mask type (*F*(2, 32) = 16.3, *p* < 0.001) and noise type (*F*(1, 16) = 132.5, *p* < 0.001). We found no interaction between mask type and noise type (*F*(2, 32) = 1.5, *p* > 0.1). Post-hoc comparisons showed that a surgical mask did not affect SRT_cut_ significantly, with an increase of 0.58 dB (*SE* = 0.35, *CI* = 0.4, 1.5) on average. A further significant increase of 1.6 dB (*SE* = 0.46, *CI* = 0.4, 2.8) was measured with use of an N95 mask, resulting in a significant total increase of 2.2 dB (*SE* = 0.37, *CI* = 1.2, 3.1) across both noise signals. SRT_cut_ was lower for the ISTS (*M* = -12.6 dB, *SE* = 1.4), compared to the Olnoise male (*M* = -5.4 dB, *SE* = 0.81) across all mask types.

**Fig 4:**
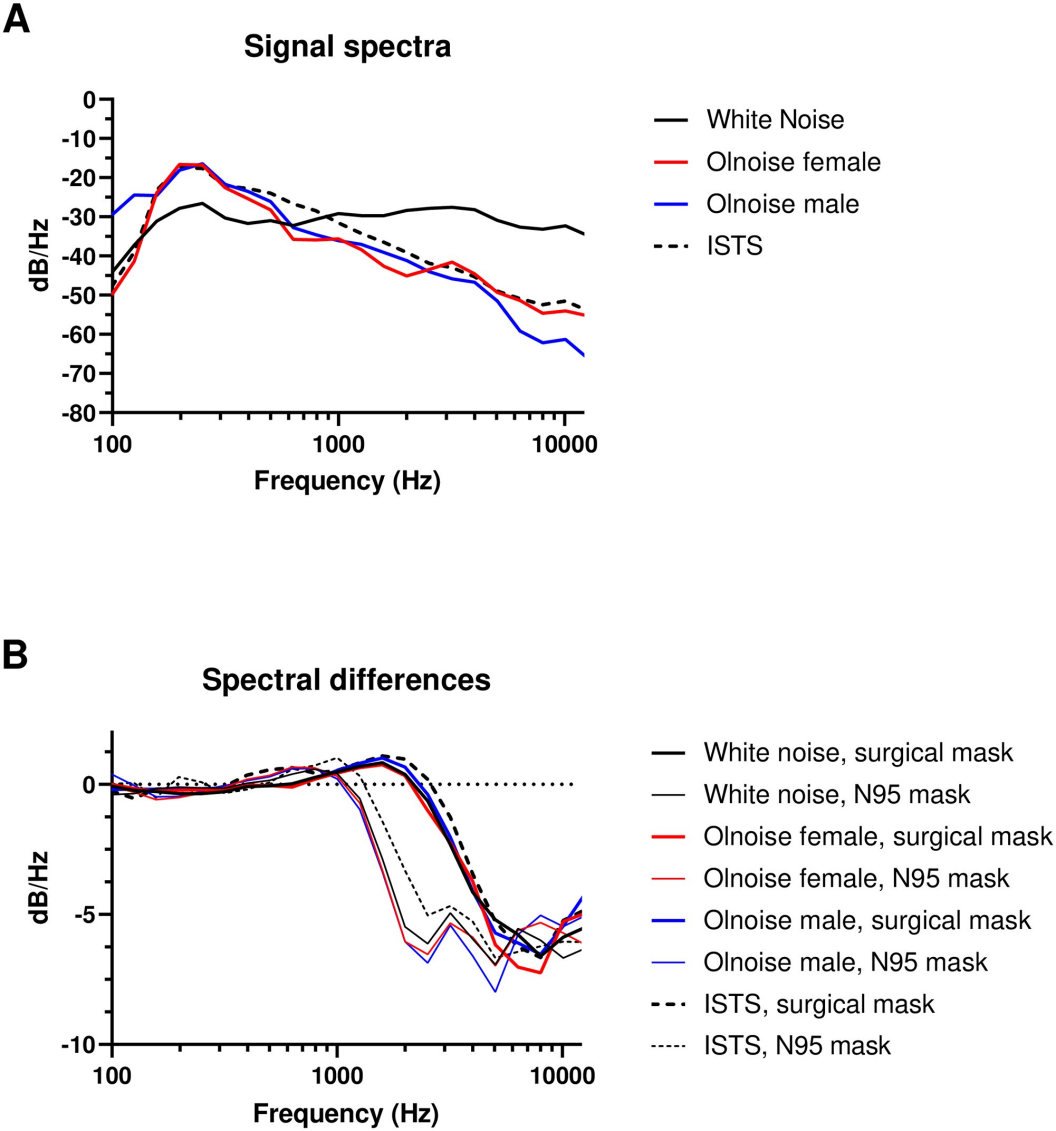
A. 1/3-octave amplitude spectra of the dummy head recordings for white noise (black line), ISTS (dashed line), Olnoise female (red line), and Olnoise male (blue line) noise signals presented without a mask. B. Spectral differences in recordings with a surgical mask (bold lines) or an N95 mask (thin lines) for all used noise signals compared with the unmasked recordings. At frequencies above 1 kHz, some mask tissue reduced the amplitudes by up to 8 dB. A surgical mask reduced the amplitudes at frequencies above 2 kHz, but a reduction was already measurable at frequencies already above 1 kHz for the N95 mask.

Fig 4A shows the 1/3-octave amplitude spectra of the dummy head recording for white noise, ISTS, Olnoise female, and Olnoise male noise signals presented without a mask. Fig 4B shows the differences between recordings without a mask and with a surgical mask or an N95 mask for all used noise signals. The amplitude reduction was comparable among all noise signals. At low frequencies below 1 kHz, no reduction was observed. At frequencies above 1 kHz, the mask tissue reduced the amplitudes by up to 8 dB. Although a surgical mask reduced the amplitudes at frequencies above 2 kHz, a reduction was already measurable at frequencies above 1 kHz with the N95 mask. For the surgical mask, maximum amplitude reduction was observed at 8 kHz. Amplitude reduction with an N95 mask showed two local maxima in 1/3-octave frequency bands at 2.520 Hz and at 5.040 Hz. A surgical mask reduced the RMS of the white noise dummy head recordings by 2.6 dB and the N95 mask reduced the RMS by 4.0 dB. Only limited RMS reduction (< 0.2 dB) was measured for all speech-noise signals.

## Discussion

We found that speech perception in noise was significantly reduced if a medical face mask was placed between the speech source and the listener. Reductions were even steeper with the use of an N95 mask. This effect is comparable to the reductions reported by Goldin et al. [11], Branda [12], and Bandaru et al. [15]. Those studies used live voice, whereas studies using audio-only recordings made with medical masks showed no significant effects on speech intelligibility [6, 16, 17].

The current findings indicate that a face mask induced SRT reductions consistently across three different noise types. Without a mask between the speaker and the listener, the measured SRT was better than the normative range of the used speech-in-noise test for signal and noise both presented from the front (S_0_N_0_) [18]. Use of masks still resulted in SRTs being within that reference value range. In daily life situations, the noise source is not fixed and would potentially be in front of the listener in many scenarios. In such cases, the baseline SRT would already be worse as compared to the used configuration with noise from behind (S_0_N_180_). Because only the speech signals would be affected by a face mask in those situations, speech-in-noise perception SRTs would be even lower, i.e., worse, than measured in the present study.

The surgical and N95 masks attenuated acoustic transmission of sound. Dummy-head recordings showed RMS reductions in speech noise signals in the same low magnitude as previously reported [6, 8]. For white noise, however, our results showed a larger RMS reduction for both mask types. A more detailed analysis of the amplitude spectrum indicated no relevant amplitude reduction for low frequencies but a pronounced reduction for higher frequencies. Both mask types modified sound transmission like a low-pass filter with cut-off frequencies of 2 kHz for the surgical mask and 1 kHz for the N95 mask. The measured magnitude of the attenuation, however, was comparable between the surgical and N95 masks and in the same range as [11] or greater than previously measured [14]. Our results strengthen previous findings by providing attenuation data for currently used medical mask types. Even if acoustic attenuation is linear and independent of the specific acoustic signals, we nevertheless detected differences among the noise types and ISTS. The minor differences could be attributed to the presentation of randomly selected different parts of the recorded clinical test materials. Therefore, the ISTS signals particularly would have differed between the specific attenuation experiments.

The observed increase in the speech-perception-in-noise threshold was below the measured attenuation of sound pressure by the masks. Because SRT is based on the difference between speech and noise sound pressure levels, an attenuation of the speech signal alone would potentially increase the SRT by the same amount. The acoustic attenuation, however, was not equal across the frequency spectrum. The low, middle, and high frequencies contributed differently to speech perception, so the observed discrepancy between acoustical sound attenuation and SRT increase is plausible.

Acoustic attenuation and reduced speech perception in noise have possible implications for communications in daily life. Because normative reference data are available for the speech-in-noise tests we used, our findings allow for a quantification of the effect. In listeners with normal hearing, the slope of the speech intelligibility function at the SRT is 17.1%/dB [18]. If the observed SRT shift of 1.58 dB (surgical mask) or 2.69 dB (N95 mask) were caused only by a mask-related speech level reduction, the estimated speech intelligibility decrease would be 27 percentage points for the surgical mask and 46 percentage points for an N95 mask used with that specific constant SNR.

The results show a greater listening effort in noise if an N95 face mask is placed between the speaker and the listener. The face masks increased the absolute threshold (SNR_cut_) of the hearing effort function, which reflects increased listening effort. The observed effect of the masks on listening effort was of the same magnitude as the speech perception threshold in noise. Low-pass filtering, such as that caused by face masks, would be expected to reduce the audibility of high-frequency portions of spoken speech, such as consonants and sibilants, or at least to increase the listening effort. Face masks increase the effort that the speaker must make, as well, with symptoms of vocal fatigue, discomfort, and coordination of speech and breathing [30]. To the listener, an increased communication effort would therefore be expected because of the face masks and alter the interpersonal components of communication.

In the present study, the face mask types were visible to the listeners, which could have biased their speech perception performance. The reported acoustic measurements, however, showed a clear modification of speech signals, which can explain the observed subjective results. An influence of habituation and training on the SRT of the matrix test we used was reduced by randomizing the experimental conditions and starting with training lists. In daily life, the sight of a speaker who uses a face mask could potentially increase the subjective perceived listening effort because of the missing lip reading and other visual, e.g. emotional cues of face-to-face communication. The results of this study, however, showed no evidence for an increased listening effort because of the masks alone.

## Conclusions

We conclude that face masks modify acoustic features of speech signals and significantly reduce speech perception in different noise signals. In combination with an additional reduction because of missing lip reading, the combined effect of face masks would be expected to have a relevant impact on daily life communication.

## Supporting information

S1 Table Individual test results. (XLSX)
